# Development and Internal Validation of a Multivariable Prediction Model for Complications After External Cephalic Version in Singleton Term Pregnancies

**DOI:** 10.3390/jcm15166443

**Published:** 2026-08-20

**Authors:** Rosa María Gallego-Pozuelo, Inmaculada Concepción Delgado-Gonzálvez, Miriam Isabel García-Pérez, Estrella Naranjo-Díaz, Olivia Varó-Torrecillas, Clara Cámara-Cases, Margarita González-Guillén, Romina Sol Liandro, Alberto Rafael Guijarro-Campillo, Catalina de Paco-Matallana, Javier Sánchez-Romero

**Affiliations:** 1 Department of Obstetrics and Gynecology, ‘Virgen de la Arrixaca’ University Hospital, 30120 Murcia, Spain; rosamaria.gallego@um.es (R.M.G.-P.); inmadelgadog@gmail.com (I.C.D.-G.); mirandemiriam@gmail.com (M.I.G.-P.); estrella12naranjo@gmail.com (E.N.-D.); oliviavaro@gmail.com (O.V.-T.); clara.camara.cc@gmail.com (C.C.-C.); margarita95mgg@gmail.com (M.G.-G.); rsliandro@gmail.com (R.S.L.); rosagallego-96@hotmail.com (A.R.G.-C.); 2 Department of Obstetrics and Gynecology, Pediatrics and Surgery, University of Murcia, 30120 Murcia, Spain; 3 Maternal-Fetal Medicine, Reproduction and Gynecology Research Group, Biomedical Research Institute of Murcia Pascual Parrilla–IMIB, 30120 Murcia, Spain; 4 Spanish Network in Maternal, Neonatal, Child and Developmental Health Research (RICORS-SAMID, RD24/0013/0018), Instituto de Salud Carlos III, 28040 Madrid, Spain

**Keywords:** breech presentation, fetal version, complications, cesarean section

## Abstract

**Objectives**: This study aimed to describe the incidence and clinical spectrum of external cephalic versión (ECV)-related complications, to evaluate pre-procedural predictors of their occurrence, and to develop and internally validate a prediction model for ECV-related complications. **Methods**: This single-center cohort study included ECV procedures performed in singleton pregnancies with non-cephalic presentation at term between January 2014 and December 2025. All procedures followed a standardized protocol, including tocolysis and either deep sedation or spinal anesthesia. ECV-related complications were defined as maternal or fetal adverse events occurring during the procedure or within 24 h after ECV. A prespecified multivariable logistic regression model was developed using clinically relevant pre-procedural variables. Model discrimination was assessed using the area under the receiver operating characteristic curve (AUC), and internal validation was performed by bootstrap resampling. **Results**: A total of 1050 ECV procedures were included, of which 733 (69.7%) were successful. Complication status was available for 1050 procedures, among which 113 ECV-related complications were recorded (10.8%). Complications were more frequent after failed than successful ECV (17.6% vs. 7.8%). The most frequent events were non-reassuring fetal heart rate patterns, major vaginal bleeding, spotting, and uterine contractions. Emergent cesarean delivery within 24 h occurred in 72 complication cases (63.7%). No neonatal deaths occurred among cases with ECV-related complications. In the final multivariable model, lower maternal body mass index (adjusted OR 0.92 per kg/m^2^), lower deepest vertical pocket (adjusted OR 0.69 per 10 mm), and breech rather than transverse lie (adjusted OR 0.24) were independently associated with ECV-related complications. Adding deepest vertical pocket significantly improved model discrimination from an AUC of 0.645 to 0.714. Bootstrap internal validation demonstrated limited optimism, with an optimism-corrected AUC of 0.699. **Conclusions**: ECV performed under a standardized protocol achieved a high success rate with an acceptable safety profile, although approximately one in ten procedures was associated with an ECV-related complication when broadly defined. Lower maternal BMI, reduced amniotic fluid volume, and breech presentation were the main pre-procedural predictors of complications. These findings may facilitate individualized counselling and procedural planning, although external validation is required before clinical implementation.

## 1. Introduction

Non-cephalic presentation at term, most commonly breech presentation, occurs in approximately 3–4% of singleton pregnancies and remains an important contributor to cesarean delivery [1,2]. In contemporary obstetric practice, many women with persistent breech presentation are delivered by cesarean section, partly because planned vaginal breech birth has been associated with higher perinatal risk in selected settings and because clinical experience in vaginal breech delivery has decreased over recent decades [3,4]. External cephalic version (ECV) is therefore a key intervention for reducing the number of fetuses in non-cephalic presentation at birth and may contribute to lowering cesarean delivery rates related to malpresentation. Current guidelines recommend offering ECV to eligible women with a singleton fetus in non-cephalic presentation at term when no contraindications are present and appropriate facilities for fetal monitoring and urgent delivery are available [5].

ECV at term reduces both non-cephalic presentation at birth and cesarean delivery, without clear evidence of worse neonatal outcomes [6]. Reported success rates vary widely between studies, reflecting differences in parity, fetal presentation, placental location, amniotic fluid volume, maternal body mass index, use of tocolysis or neuraxial analgesia, deep sedation and operator experience [5,7]. In clinical practice, this variability is relevant because the decision to attempt ECV is usually made after counselling the patient about both the expected probability of success and the potential risks of the procedure.

ECV is generally considered a safe procedure, and severe complications are uncommon. A large meta-analysis of nearly 13,000 ECV attempts reported an overall complication rate of 6.1%, a serious complication rate of 0.24%, and an emergency cesarean delivery rate of 0.35% [7]. Reported adverse events include transient fetal heart rate abnormalities, vaginal bleeding, placental abruption, premature rupture of membranes, umbilical cord prolapse, fetomaternal hemorrhage, and the need for urgent cesarean delivery [2,5]. Transient fetal bradycardia during or shortly after ECV has been described relatively frequently, although in most cases it is self-limited and does not result in adverse neonatal outcome [8]. Nevertheless, even infrequent complications may have immediate clinical consequences, which explains why ECV should be performed in settings where fetal assessment, obstetric intervention, and emergency cesarean delivery are available [5]. Emergency cesarean delivery and neonatal outcomes should be interpreted as downstream clinical consequences of ECV-related complications rather than independent validation outcomes of the prediction model.

Most published studies on ECV have focused on procedural success, delivery mode after attempted version, or neonatal outcomes [9,10,11]. Several models have been developed to predict ECV success using maternal, fetal, and ultrasound-related variables such as parity, maternal body mass index, placental location, estimated fetal weight, amniotic fluid volume, and type of malpresentation [9,12]. However, less attention has been paid to the prediction of ECV-related complications. This distinction is clinically important because the factors associated with a successful version may not be the same as those associated with procedural risk. Although large cohorts have described the frequency and type of ECV-related complications, multivariable analyses specifically evaluating pre-procedural predictors of these events remain limited [11,13].

Identifying maternal, fetal, and ultrasound characteristics associated with ECV-related complications could help clinicians provide more individualized counselling and improve procedural planning. This may be particularly useful in women with borderline clinical conditions, reduced amniotic fluid, less favorable fetal position, previous uterine surgery, or other factors that may influence the risk-benefit balance of attempting ECV. The objective of the present study was therefore to describe the incidence and clinical spectrum of complications following ECV in a large cohort of singleton pregnancies with non-cephalic presentation at term, to evaluate pre-procedural factors associated with the occurrence of ECV-related complications, and to develop and internally validate a prediction model for ECV-related complications.

## 2. Materials and Methods

This was a single-center cohort study conducted at a tertiary university hospital in Spain between 1 January 2014 and 31 December 2025. The study was based on the same institutional external cephalic version (ECV) database previously used to evaluate ECV outcomes under a standardized protocol combining tocolysis with deep sedation or spinal anesthesia [11]. The present analysis extends the cohort through December 2025, adding 61 procedures and resulting in a total of 1051 ECV attempts. The database comprised a retrospective component including all ECV procedures performed between January 2014 and May 2020, and a prospective component initiated in June 2020. Prospective recruitment has continued thereafter using the same predefined variables, procedural definitions, and outcome criteria to ensure consistency in data collection throughout the study period.

The present analysis aimed to develop and internally validate a multivariable prediction model for ECV-related complications using exclusively pre-procedural maternal, fetal, and ultrasound characteristics that could be available before attempting the procedure. The study was approved by the Institutional Review Board of the ‘Virgen de la Arrixaca’ University Hospital (approval code: 2020-5-6-HCUVA), and was conducted in accordance with the principles of the Declaration of Helsinki. Written informed consent was obtained from prospectively recruited participants, and all data were anonymized before analysis. The Institutional Review Board approved the retrospective use of anonymized clinical data collected before study initiation in accordance with local regulations.

### 2.1. Participants

All women undergoing attempted ECV for singleton non-cephalic presentation at term during the study period were eligible for inclusion. ECV was offered from 36 weeks of gestation to women with breech presentation or transverse/oblique lie in the absence of contraindications to vaginal delivery. Before the procedure, obstetric history and ultrasound findings were reviewed to confirm fetal presentation, fetal biometry, placental location, and amniotic fluid volume. External cephalic version was routinely scheduled and performed from 37 + 0 weeks onwards according to the institutional protocol.

Women were considered ineligible for ECV in the presence of severe pre-eclampsia, confirmed rupture of membranes, anhydramnios or severely reduced amniotic fluid, recent vaginal bleeding, placenta previa, or any absolute indication for cesarean delivery. Cases in which spontaneous cephalic version occurred before the planned procedure, women who entered active labor before ECV, and those who declined the procedure were not included among attempted ECV procedures.

### 2.2. ECV Protocol

All ECV procedures were performed according to a standardized institutional protocol [14]. Participants fasted for at least 8 h before the procedure. ECV was performed in an operating room by a dedicated multidisciplinary team including experienced obstetricians, a midwife, and an anesthesiologist. Maternal vital signs were monitored throughout the procedure, including non-invasive blood pressure, heart rate, electrocardiography, and oxygen saturation.

Tocolysis was administered before the procedure using ritodrine at a dose of 0.2 mg/min for 30 min. The anesthetic approach was determined according to anesthesiologist criteria and clinical characteristics, and consisted of either deep sedation with propofol or spinal anesthesia with bupivacaine and intrathecal fentanyl. Women were placed in a mild Trendelenburg position, and ECV was attempted using the forward-roll technique. A maximum of two attempts were performed.

Fetal presentation was reassessed by ultrasound immediately after the procedure. Fetal well-being was monitored by cardiotocography after ECV, and additional fetal assessment was performed according to the institutional protocol. Rhesus-negative women received anti-D immunoglobulin.

### 2.3. Outcomes

The primary outcome of the present analysis was the occurrence of ECV-related complications. ECV-related complications were defined as maternal or fetal adverse events occurring during the procedure or within the first 24 h after ECV. Recorded complications included non-reassuring fetal heart rate, major vaginal bleeding, spotting, uterine contractions, premature rupture of membranes, cord prolapse, and bronchoaspiration. ECV-related complications were classified as minor (spotting, transient uterine contractions, and prelabor rupture of membranes) or major (major vaginal bleeding suspicious for placental abruption, umbilical cord prolapse, bronchoaspiration, and non-reassuring fetal heart rate pattern requiring clinical intervention), according to the classification used in our previous publications. For the primary analyses, both categories were combined into a composite outcome representing any ECV-related complication. No blinding of outcome assessment was performed because complications were assessed during routine clinical care at the time of the procedure.

Major vaginal bleeding was defined as visible blood loss greater than 50 mL or any episode raising clinical suspicion of placental abruption. Spotting was defined as vaginal blood loss below 50 mL. Uterine contractions were defined as regular painful uterine activity after ECV without associated cervical change (2–3 contractions every 10 min sustained for at least 1 h). Non-reassuring fetal heart rate was classified according to institutional criteria [15] based on abnormal cardiotocographic patterns, including persistent fetal bradycardia (<100 bpm lasting >7 min) and recurrent or prolonged decelerations requiring clinical intervention, such as prolonged fetal monitoring, hospital admission, or emergency cesarean delivery. Prelabor rupture of membranes was defined as leakage of amniotic fluid confirmed by clinical examination or biochemical testing. Bronchoaspiration was defined as aspiration of gastric contents during the anesthetic procedure.

Each procedure was assigned a single complication category corresponding to the complication considered clinically most relevant. Although more than one clinical finding could occasionally occur during the same procedure, only the principal complication was recorded in the study database to avoid double counting. Consequently, each patient contributed only one complication to the analyses.

ECV success was defined as cephalic presentation immediately after the procedure. Only variables available before the procedure were considered candidate predictors for model development, ensuring that the final model could be used for pre-procedural risk estimation. Delivery and neonatal outcomes were also collected, including gestational age at delivery, mode of delivery, emergent cesarean delivery within 24 h after ECV, neonatal birthweight, umbilical artery pH, Apgar scores, neonatal admission, neonatal intensive care unit admission, and neonatal death.

### 2.4. Statistical Analysis

Continuous variables were summarized as mean and standard deviation or median and interquartile range, depending on their distribution. Categorical variables were expressed as absolute and relative frequencies. Comparisons between women with and without ECV-related complications were performed using Student’s *t*-test or Welch’s *t*-test for continuous variables, according to variance assumptions, and Pearson’s chi-square test or Fisher’s exact test for categorical variables, as appropriate.

Pre-procedural variables evaluated as potential predictors of ECV-related complications included maternal age, parity, previous cesarean delivery, body mass index, gestational age at ECV, estimated fetal weight, placental location, fetal position, deepest vertical pocket, type of analgesia or anesthesia, and maternal comorbidity. Only variables considered clinically relevant before the procedure were eligible for inclusion in the prediction models.

Multivariable analyses were performed using a complete-case approach. Only women with complete information for all candidate predictors included in each model were analyzed. A prespecified multivariable logistic regression model including gestational age at ECV, maternal BMI, estimated fetal weight, fetal presentation, and deepest vertical pocket was considered the primary prediction model because all variables were available before the procedure and were selected according to clinical relevance, previous evidence, and their availability before the ECV procedure. Automated variable-selection procedures were intentionally avoided. Four prespecified nested logistic regression models were subsequently developed to evaluate the incremental predictive value of additional clinically relevant variables. Model 1 included gestational age at ECV, maternal BMI, estimated fetal weight, and fetal presentation. Model 2 additionally included deepest vertical pocket and was designated as the primary model. Model 3 further included previous cesarean delivery, and Model 4 additionally included placental location; Models 3 and 4 were considered sensitivity analyses.

Estimated fetal weight was scaled per 100 g and deepest vertical pocket per 10 mm to improve the clinical interpretability of adjusted odds ratios. Model assumptions were assessed before final interpretation. Linearity in the logit was assessed using graphical inspection of smoothed plots of each continuous predictor against the logit of the outcome, and collinearity was assessed using variance inflation factors. Results were expressed as adjusted odds ratios with 95% confidence intervals and *p* values.

Model discrimination was evaluated using receiver operating characteristic curves and the area under the curve with 95% confidence intervals. AUCs were compared using DeLong’s test for paired ROC curves. Additional pairwise DeLong comparisons, likelihood ratio tests between nested models, and goodness-of-fit indices were reported as supplementary analyses. Model fit was assessed using the Akaike information criterion, Bayesian information criterion, Brier score, and the Hosmer–Lemeshow goodness-of-fit test.

Internal validation of the final model (Model 2) was performed with 1000 bootstrap resamples. Optimism-corrected estimates of discrimination (AUC), calibration intercept, calibration slope, and Brier score were obtained. Calibration was graphically assessed using bootstrap-corrected calibration plots. Sensitivity analyses were performed by additionally incorporating previous cesarean delivery (Model 3) and placental location (Model 4) into the final model.

All statistical tests were two-sided, and statistical significance was set at *p* < 0.05. Statistical analyses and table generation were performed using Stata 19 BE Edition (StataCorp, College Station, TX, USA) and RStudio version 2026.06.0+242 with R version 4.6.1.

## 3. Results

### 3.1. Study Population

**Figure 1:**
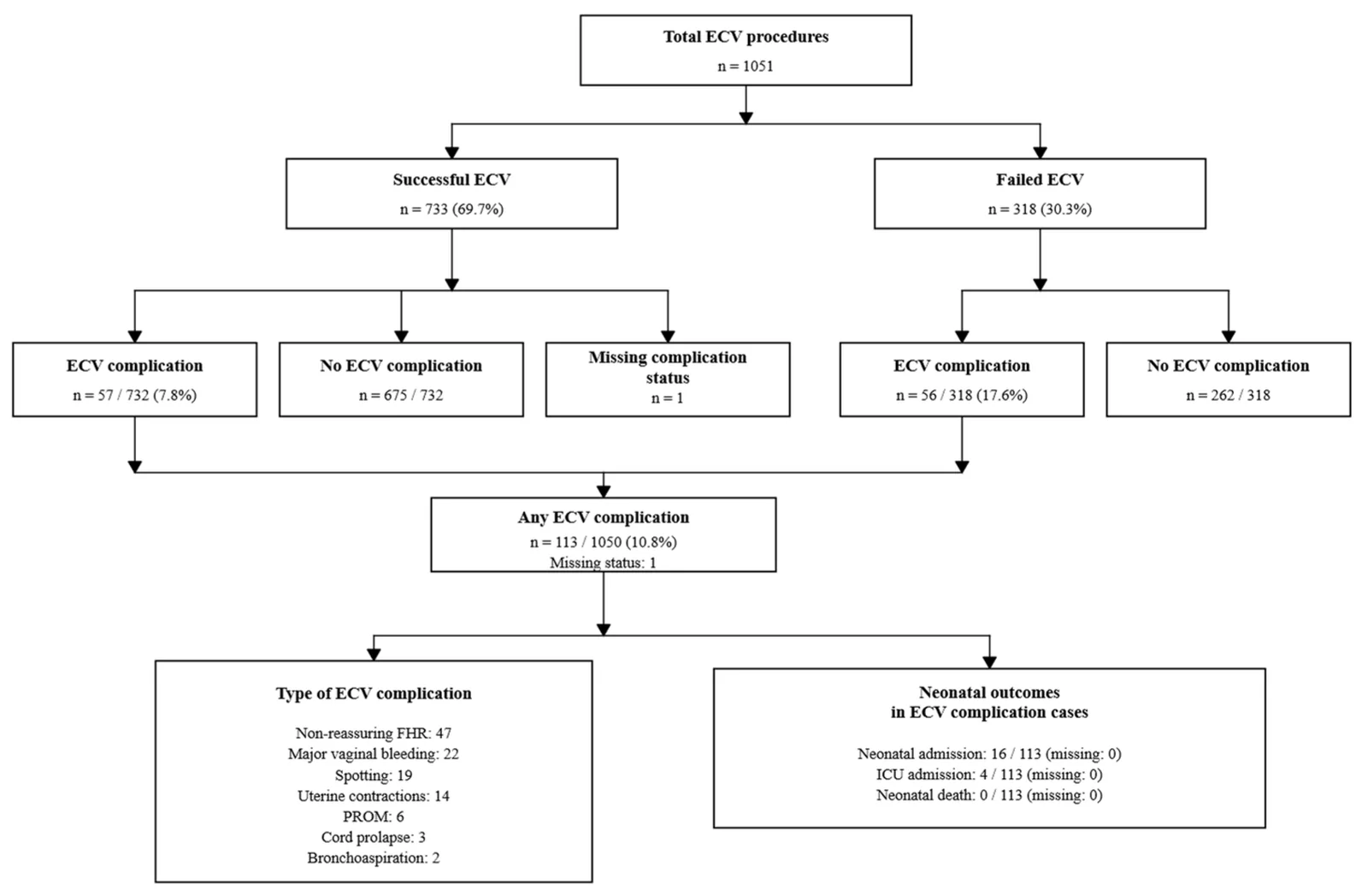
Flowchart of external cephalic version procedures according to ECV outcome, occurrence of ECV-related complications, type of complication, and neonatal outcomes in ECV complication cases.

A total of 1051 ECV procedures were included in the analysis. ECV was successful in 733 cases (69.7%) and failed in 318 cases (30.3%). Information on ECV-related complications was available for 1050 procedures, as one case had missing complication status. Overall, 113 ECV-related complications were recorded, corresponding to a complication rate of 10.8% (113/1050). Complications were observed in 57 of 732 successful ECV procedures (7.8%) and in 56 of 318 failed ECV procedures (17.6%). Among the 113 ECV-related complications, 39 were classified as minor and 74 as major, corresponding to 3.7% and 7.0% of the 1050 evaluable ECV procedures, respectively (Table 1). The flow of patients according to ECV outcome, occurrence of complications, type of complication, and neonatal outcomes among cases with ECV complications is shown in Figure 1.

Baseline and procedural characteristics according to ECV complication status are summarized in Table 2. Maternal age, gestational age at ECV, estimated fetal weight, parity, previous cesarean delivery, placental location, use of analgesia or anesthesia, and maternal comorbidity were similar between groups. However, women with ECV-related complications had a lower mean BMI than those without complications (24.92 ± 3.78 vs. 26.94 ± 5.03 kg/m^2^, *p* < 0.001) and a lower DVP (42.90 ± 12.78 vs. 51.77 ± 16.80 mm, *p* < 0.001). Accordingly, a DVP below 30 mm was more frequent among cases with complications (14.3% vs. 6.1%, *p* = 0.006). Breech presentation was also more frequent in the complication group (98.2% vs. 89.6%, *p* = 0.001), whereas transverse lie was less frequent. ECV failure was significantly more common among cases with complications than among those without complications (49.6% vs. 28.0%, *p* < 0.001). Maternal comorbidities were infrequent and were similarly distributed between groups. The “other” category included rare conditions such as Chagas disease, maternal HIV infection with undetectable viral load, maternal hepatitis B, Arnold–Chiari syndrome, maternal myopathy, and Crohn disease.

Among the 113 ECV-related complications, the most frequent event was non-reassuring fetal heart rate, observed in 47 cases. This was followed by major vaginal bleeding in 22 cases, spotting in 19 cases, uterine contractions in 14 cases, premature rupture of membranes in 6 cases, cord prolapse in 3 cases, and bronchoaspiration in 2 cases.

### 3.2. Delivery and Neonatal Outcomes

Delivery and neonatal outcomes according to ECV complication status are presented in Table 3. Cases with ECV-related complications delivered at an earlier mean gestational age than those without complications (37.72 ± 0.94 vs. 39.60 ± 1.09 weeks, *p* < 0.001), and neonatal birthweight was lower in the complication group (2925.84 ± 350.74 vs. 3241.31 ± 435.76 g, *p* < 0.001). Umbilical artery pH was also lower among cases with complications (7.25 ± 0.10 vs. 7.28 ± 0.07, *p* = 0.001), as were Apgar scores at 1 min (8.19 ± 1.51 vs. 8.86 ± 0.67, *p* < 0.001) and 5 min (9.55 ± 0.91 vs. 9.90 ± 0.54, *p* < 0.001).

Emergent cesarean delivery within 24 h after ECV occurred in 72 of 113 cases with complications (63.7%), whereas no such cases were recorded among women without complications. Low Apgar score at 1 min was more frequent among neonates born after ECV-related complications (23.0% vs. 3.6%, *p* < 0.001), whereas the frequency of umbilical artery pH ≤ 7.00 and Apgar score at 5 min ≤ 7 did not differ significantly between groups.

Neonatal admission was more frequent in the complication group than in the non-complication group (14.2% vs. 2.1%, *p* < 0.001), as was neonatal ICU admission (3.5% vs. 0.5%, *p* = 0.011). Among neonates admitted after ECV-related complications, the most frequent reasons for admission were transient tachypnea and neonatal respiratory distress syndrome. The four neonatal ICU admissions in the complication group were due to transient tachypnea or respiratory distress syndrome.

Among the 113 cases with ECV-related complications, neonatal admission occurred in 16 cases, ICU admission in 4 cases, and no neonatal deaths were observed. No intrauterine fetal demises or neonatal deaths occurred among cases with ECV-related complications. In the non-complication group, two intrauterine fetal demises and one neonatal death were recorded. One intrauterine fetal death occurred 12 days after a successful ECV following prelabor rupture of membranes and umbilical cord prolapse, and the second occurred 7 days after a failed ECV for an unknown cause. The neonatal death occurred in an infant with prenatally diagnosed Loucks-Innes syndrome, tetralogy of Fallot, and vermian agenesis after prolonged neonatal intensive care admission. None of these events occurred within the predefined 24 h window for ECV-related complications.

### 3.3. Development of the Prediction Model

The prespecified multivariable prediction model included 876 complete cases with 97 ECV-related complications. Lower maternal BMI, lower deepest vertical pocket, and breech rather than transverse presentation were independently associated with ECV-related complications (Supplementary Table S1).

**Figure 2:**
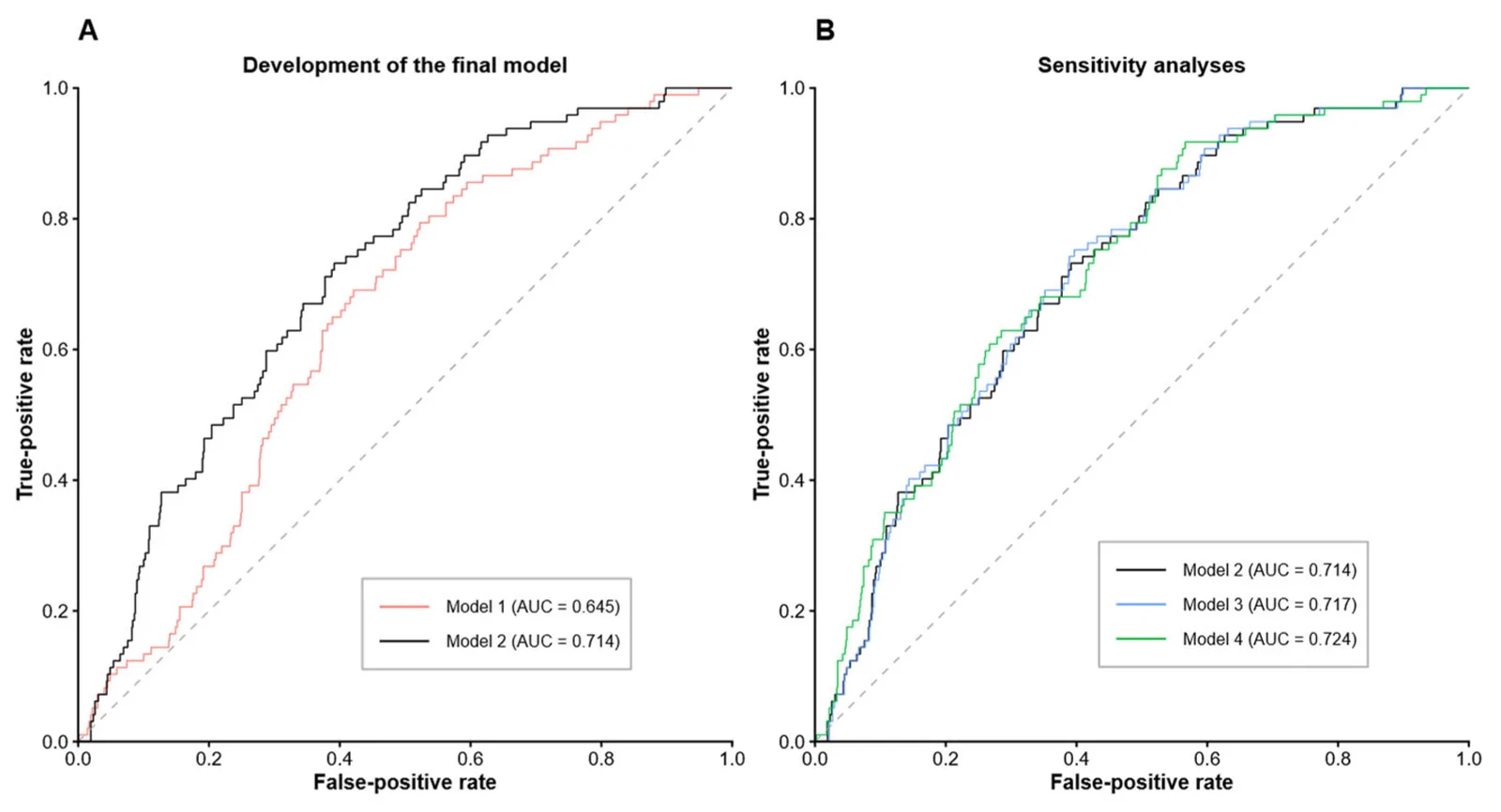
Receiver operating characteristic curves of the primary prediction model of ECV-related complications and sensitivity analyses. (**A**) Development of the final model: Model 1 included gestational age at ECV, BMI, estimated fetal weight, and fetal position; Model 2 additionally included the deepest vertical pocket and was designated as the final prediction model. (**B**) Sensitivity analyses: Model 3 additionally included previous cesarean delivery, and Model 4 additionally included placental location. AUC, area under the curve; BMI, body mass index; ECV, external cephalic version. AUC, area under the curve; ECV, external cephalic version.

Adding deepest vertical pocket significantly improved discrimination compared with the reference model (AUC 0.645 vs. 0.714; DeLong *p* = 0.002), whereas inclusion of previous cesarean delivery and placental location did not materially improve predictive performance (Figure 2). Likelihood ratio testing also supported inclusion of deepest vertical pocket in the final model (*p* < 0.001) (Table 4).

### 3.4. Sensitivity Analyses

Sensitivity analyses were performed by adding previous cesarean delivery (Model 3) and placental location (Model 4) to the final model. Maternal BMI, deepest vertical pocket, and fetal presentation remained independently associated with ECV-related complications in both models. Previous cesarean delivery was not significantly associated with complications (adjusted OR 1.55, 95% CI 0.51–3.91), and placental location was not associated with improved model performance. Model discrimination changed only minimally (AUC 0.717 and 0.724, respectively) (Figure 2B and Supplementary Table S2).

### 3.5. Internal Validation

**Figure 3:**
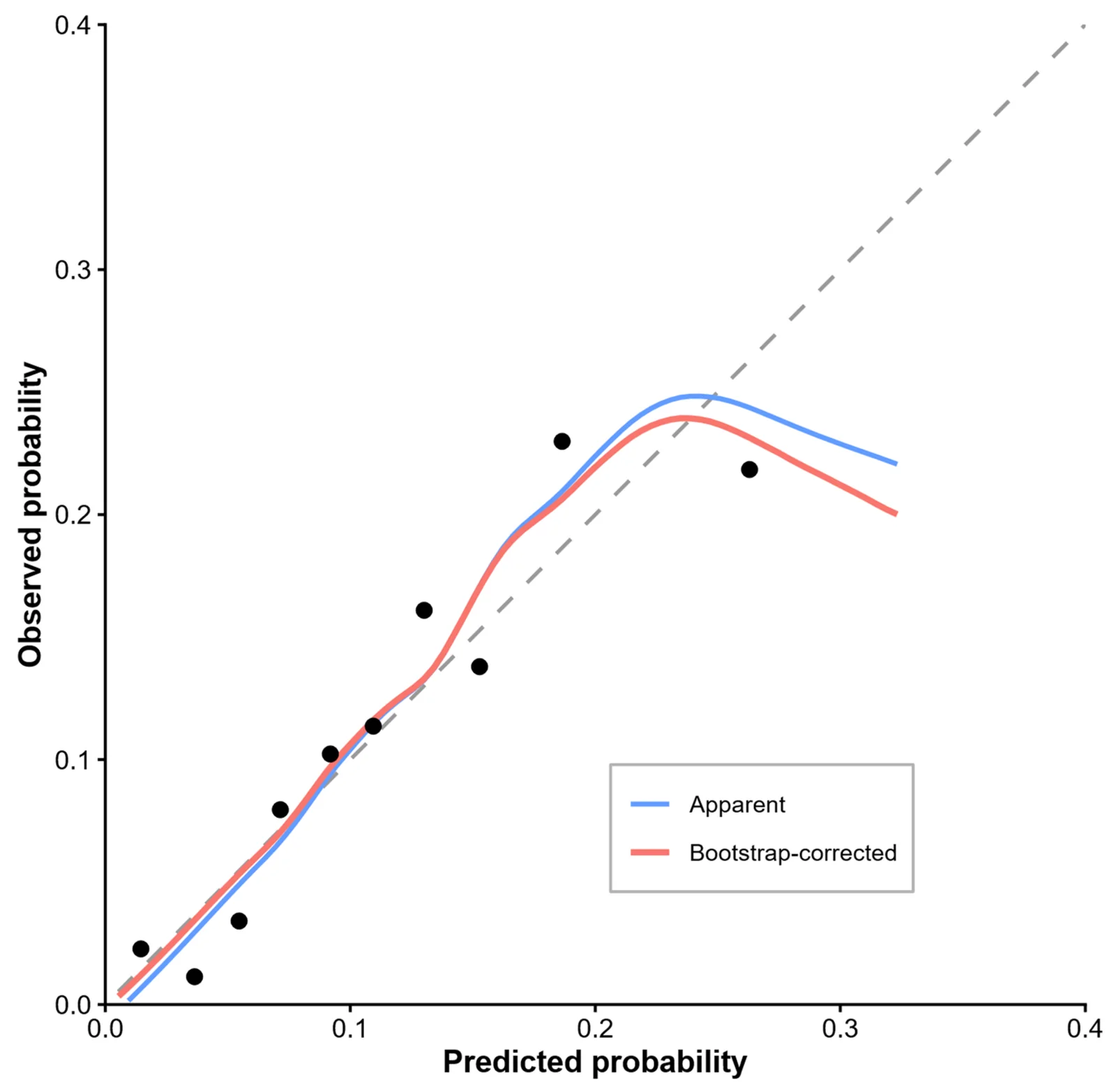
Bootstrap calibration plot of the final prediction model for ECV-related complications. The blue line represents the apparent calibration and the red line the optimism-corrected calibration obtained from 1000 bootstrap resamples. The dashed line represents perfect calibration.

Internal bootstrap validation demonstrated limited optimism of the final model. Bootstrap internal validation yielded an optimism-corrected AUC of 0.699, a calibration slope of 0.836, a calibration intercept of −0.308, and an optimism-corrected Brier score of 0.095. The bootstrap-corrected calibration plot showed good agreement between predicted and observed probabilities across most of the prediction range (Figure 3 and Supplementary Table S3).

## 4. Discussion

In this large single-center cohort of 1051 ECV procedures performed under a standardized protocol with systematic tocolysis and either deep sedation or spinal anesthesia, ECV was successful in 69.7% of cases. ECV-related complications were recorded in 10.8% of evaluable procedures. Most complications consisted of non-reassuring fetal heart rate patterns or bleeding-related events, whereas severe adverse events such as cord prolapse and bronchoaspiration were uncommon. Complications were more frequent after failed ECV than after successful ECV, and emergent cesarean delivery within 24 h was required in 63.7% of cases with complications. Neonatal admission and neonatal ICU admission were more frequent among cases with ECV-related complications, but no neonatal deaths occurred in this group.

The multivariable analysis identified lower BMI, lower deepest vertical pocket, and breech presentation rather than transverse lie as the pre-procedural variables most consistently associated with ECV-related complications. The addition of deepest vertical pocket to the base model produced the largest improvement in model discrimination, increasing the AUC from 0.645 to 0.714. The prespecified primary model included gestational age, BMI, estimated fetal weight, fetal presentation, and DVP. The absence of meaningful improvement after adding previous cesarean delivery or placental location supported retaining this more parsimonious specification. Bootstrap internal validation showed only limited optimism, supporting the stability of the final model despite its moderate discriminative performance.

The overall complication rate observed in this study was higher than the pooled rate of 6.1% reported in the meta-analysis by Grootscholten et al. and higher than the 4.7% total complication rate reported by Rodgers et al. in a tertiary hospital cohort. However, it was very similar to the 10.2% complication rate previously reported from the same institutional database [7,11,13].

Several factors may explain these differences. First, the definition of ECV-related complications varies substantially across studies. In the present analysis, complications were defined broadly and included transient non-reassuring fetal heart rate patterns, spotting, minor bleeding, uterine contractions, and other events occurring within the first 24 h after ECV. Some previous studies have restricted their analysis to serious complications or have classified transient fetal heart rate abnormalities separately. Second, all procedures in our cohort were performed in an operating room with close maternal and fetal monitoring, which may have increased detection of transient or self-limited abnormalities. Third, local thresholds for emergent cesarean delivery after ECV may differ between centers, particularly in units where the procedure is performed under anesthesia or deep sedation with immediate surgical availability.

Therefore, the 10.8% complication rate should not be interpreted as an unexpectedly high severe morbidity rate. Rather, it likely reflects a combination of broad definitions, active surveillance, and systematic recording of both minor and clinically relevant events. This distinction is important when counselling patients, because the overall complication rate includes events with very different clinical implications.

The high ECV success rate observed in our cohort was achieved using a standardized protocol combining systematic ritodrine tocolysis with either deep propofol sedation or spinal anesthesia [14]. Current evidence consistently supports the use of parenteral tocolysis to improve ECV success, whereas the role of anesthetic techniques is less straightforward [16]. Meta-analyses have shown that neuraxial anesthesia increases the likelihood of successful version, although it may also increase maternal hypotension, and its overall impact on safety remains uncertain [17,18,19]. Similarly, propofol sedation has shown promising results in observational studies, but robust comparative evidence is still limited, and ongoing randomized trials are expected to clarify its role [20,21].

These considerations are important when interpreting our findings. Strategies that facilitate fetal manipulation may improve procedural success while simultaneously increasing the detection of transient fetal heart rate abnormalities because ECV is performed under closer maternal and fetal surveillance and in settings with immediate access to emergency cesarean delivery. Consequently, a higher overall rate of recorded complications does not necessarily indicate a less favorable safety profile. In our cohort, despite the relatively broad definition of ECV-related complications, severe neonatal outcomes were uncommon, supporting the overall safety of ECV when performed within a standardized protocol and appropriate obstetric setting.

Although some neonatal outcomes, including umbilical artery pH and Apgar scores, differed significantly between groups, the magnitude of these differences was small. Although statistically significant, these differences are unlikely to be clinically meaningful.

Lower BMI was consistently associated with ECV-related complications across all models. This finding is somewhat counterintuitive, because higher BMI is usually associated with lower ECV success. The biological mechanism underlying this association is uncertain. It is possible that, in women with lower BMI, manual force may be transmitted more directly to the uterus and fetus because of reduced abdominal wall thickness. Another possibility is that lower resistance during manipulation may allow more complete or more vigorous attempts. These explanations remain speculative and should be interpreted as hypothesis-generating.

Deepest vertical pocket was one of the most relevant variables in the analysis. Lower amniotic fluid volume was associated with higher odds of complications, and adding deepest vertical pocket to the base model produced the largest improvement in discrimination. This association is clinically plausible, as reduced amniotic fluid may limit fetal mobility, increase mechanical difficulty during rotation, and favor cord compression, membrane disruption, or transient fetal heart rate abnormalities. Amniotic fluid volume has been widely studied as a predictor of ECV success; our results suggest that it may also be useful when assessing procedural risk.

Fetal position was also consistently associated with complications. Transverse lie showed lower odds of complications compared with breech presentation. This is in line with clinical experience, as transverse or oblique lie is usually more favorable for version, while breech presentation may require a more complex rotational movement, especially when the presenting part is low or engaged. Thus, fetal position appears relevant not only for estimating the probability of success but also for anticipating procedural difficulty and potential adverse events.

Previous cesarean delivery was not significantly associated with complications in this cohort. This finding is consistent with the literature suggesting that ECV after previous cesarean delivery can be performed with acceptable safety in selected patients, although sample size and patient selection remain important considerations [22,23]. Similarly, placental location was not a significant predictor of complications in the final model. Although anterior placenta may theoretically influence technical difficulty or operator caution, the association between placental location and ECV outcomes remains inconsistent across studies.

The proposed prediction model was not only developed but also internally validated using bootstrap resampling, providing optimism-corrected estimates of discrimination and calibration. The bootstrap-corrected calibration plot showed reasonable agreement between predicted and observed probabilities across most of the observed prediction range, with greater deviation at higher predicted probabilities. The model relies exclusively on variables routinely available before the procedure, making it readily applicable during pre-procedural counselling without requiring additional investigations. Although its discriminative ability is moderate rather than excellent, this level of performance is comparable to that of many clinically useful obstetric prediction models. The model should therefore be considered a tool to support individualized risk assessment and procedural planning rather than to determine whether ECV should be attempted. External validation in independent populations and evaluation of its clinical impact, including decision-analytic approaches such as Decision Curve Analysis, will be necessary before routine implementation.

This study has several strengths. It includes a large cohort of ECV procedures from a high-volume tertiary center with a standardized protocol, systematic use of tocolysis and anesthesia or deep sedation, and detailed recording of ECV-related complications. The prediction model was developed using prespecified clinically relevant predictors available before the procedure, avoiding automated variable-selection strategies, and underwent internal validation with bootstrap resampling to quantify optimism and assess model calibration. This methodological approach reduces the risk of overfitting and enhances the credibility of the reported predictive performance. The exclusive use of routinely available pre-procedural variables increases the potential applicability of the model in everyday clinical practice.

Several limitations should be acknowledged. First, this was a single-center observational study conducted in a high-volume tertiary referral center using a standardized ECV protocol; the predictive performance of the model may differ in institutions with different case-mix, operator experience, or peri-procedural management. Consequently, external validation in independent populations is essential before routine clinical implementation. Second, the observational design precludes causal inference. Third, the anesthetic technique was chosen according to clinical judgement rather than random allocation. Therefore, comparisons between deep sedation and spinal anesthesia should be interpreted with caution, as the two groups may not have been directly comparable. Fourth, the broad definition of complications may have increased the overall complication rate compared with studies using more restrictive definitions. Fifth, serious complications were uncommon, limiting the ability to identify predictors of individual severe events. Sixth, multivariable analyses were based on a complete-case approach. Although the proportion of missing data was low for most variables, missing deepest vertical pocket measurements resulted in the exclusion of some procedures, and some degree of selection bias cannot be excluded. Finally, although internal validation using bootstrap resampling demonstrated limited optimism, internal validation cannot replace external validation, which remains the reference standard for evaluating model transportability.

## 5. Conclusions

ECV performed under a standardized protocol with systematic tocolysis and either deep sedation or spinal anesthesia achieved a high success rate and an acceptable safety profile. Although complications occurred in approximately one in ten procedures when broadly defined, severe neonatal adverse outcomes were uncommon. Lower BMI, reduced deepest vertical pocket, and breech presentation were the pre-procedural factors most consistently associated with ECV-related complications. A prediction model based exclusively on routinely available pre-procedural variables showed moderate discrimination and good internal validity after bootstrap correction, supporting its potential role in individualized pre-procedural counselling and procedural planning. Nevertheless, external validation in independent populations is required before the model can be recommended for routine clinical use.

## Figures and Tables

**Table 1:** Classification and frequency of ECV-related complications. Values are presented as numbers and percentages of all recorded ECV-related complications.

Clinical Severity	ECV-Related Complication	*n*
Minor complications	Spotting	19 (16.8%)
	Uterine contractions	14 (12.4%)
	Prelabor rupture of membranes	6 (5.3%)
Minor subtotal		39 (34.5%)
Major complications	Non-reassuring fetal heart rate pattern	47 (41.6%)
	Major vaginal bleeding	22 (19.5%)
	Umbilical cord prolapse	3 (2.7%)
	Bronchoaspiration	2 (1.8%)
Major subtotal		74 (65.5%)
Total		113 (100%)

**Table 2:** Baseline and procedural characteristics according to ECV complication.

Characteristic	No ECV Complication (*n* = 937)	ECV Complication (*n* = 113)	*p*
Maternal age, years	33.12 (5.50)	32.17 (5.43)	0.084
Gestational age at ECV, weeks	37.49 (0.65)	37.44 (0.57)	0.409
Estimated fetal weight, g	2785.62 (384.90)	2710.42 (380.86)	0.052
Nulliparity	516/937 (55.1%)	69/113 (61.1%)	0.231
Previous cesarean section	44/937 (4.7%)	6/113 (5.3%)	0.814
Body mass index, kg/m^2^	26.94 (5.03)	24.92 (3.78)	<0.001
≤25 kg/m^2^	364/932 (39.1%)	61/112 (54.5%)	<0.001
25–30 kg/m^2^	343/932 (36.8%)	41/112 (36.6%)	
30–35 kg/m^2^	157/932 (16.8%)	8/112 (7.1%)	
>35 kg/m^2^	68/932 (7.3%)	2/112 (1.8%)	
Placental location			0.105
Anterior	520/937 (55.5%)	70/113 (61.9%)	
Posterior	343/937 (36.6%)	30/113 (26.5%)	
Uterine fundus	20/937 (2.1%)	5/113 (4.4%)	
Lateral wall	54/937 (5.8%)	8/113 (7.1%)	
Deepest Vertical Pocket (DVP), mm	51.77 (16.80)	42.90 (12.78)	<0.001
≤30 mm	48/786 (6.1%)	14/98 (14.3%)	0.006
>30 mm	738/786 (93.9%)	84/98 (85.7%)	
Fetal position			0.001
Breech	840/937 (89.6%)	111/113 (98.2%)	
Transverse lie	97/937 (10.4%)	2/113 (1.8%)	
Analgesia/anesthesia			0.096
No	13/937 (1.4%)	0/113 (0.0%)	
Sedation	860/937 (91.8%)	100/113 (88.5%)	
Spinal anesthesia	64/937 (6.8%)	13/113 (11.5%)	
Successful ECV	675/937 (72.0%)	57/113 (50.4%)	<0.001
Maternal comorbidity			0.857
GDM	53/929 (5.7%)	7/113 (6.2%)	
Pregestational DM	13/929 (1.4%)	0/113 (0.0%)	
Cholestasis	2/929 (0.2%)	0/113 (0.0%)	
Gestational Hypertension	4/929 (0.4%)	0/113 (0.0%)	
Preeclampsia	7/929 (0.8%)	1/113 (0.9%)	
Others	5/929 (0.5%)	1/113 (0.9%)	

**Table 3:** Delivery and neonatal outcomes according to ECV complication. ICU: Intensive Care Unit. NC: Not comparable.

Outcome	No ECV Complication (*n* = 937)	ECV Complication (*n* = 113)	*p*
Gestational age at delivery, weeks	39.60 (1.09)	37.72 (0.94)	<0.001
Birthweight, g	3241.31 (435.76)	2925.84 (350.74)	<0.001
Neonatal admission	19/924 (2.1%)	16/113 (14.2%)	<0.001
Neonatal ICU admission	5/924 (0.5%)	4/113 (3.5%)	0.011
Intrauterine fetal demise	2/924 (0.2%)	0/113 (0.0%)	NC
Neonatal death	1/924 (0.1%)	0/113 (0.0%)	NC
Emergent CS 24 h after ECV	0/928 (0.0%)	72/113 (63.7%)	NC
Umbilical artery pH	7.28 (0.07)	7.25 (0.10)	0.001
Umbilical artery pH ≤ 7.00	4/737 (0.5%)	2/100 (2.0%)	0.154
Apgar score at 1st minute	8.86 (0.67)	8.19 (1.51)	<0.001
Apgar score at 1st minute ≤ 7	33/921 (3.6%)	26/113 (23.0%)	<0.001
Apgar score at 5th minute	9.90 (0.54)	9.55 (0.91)	<0.001
Apgar score at 5th minute ≤ 7	9/921 (1.0%)	3/113 (2.7%)	0.135

**Table 4:** Development and incremental performance of the prediction models for ECV-related complications. AUC values were estimated from receiver operating characteristic curves. All models were fitted on the same complete-case sample (*n* = 876 and 97 events). AUCs were compared against Model 1 using DeLong’s test for paired ROC curves, and for sensitivity analyses, Model 2 was considered as the reference.

Model	Added Variable	AUC (95% CI)	ΔAUC	*p*	LRT *χ*^2^ (df)	LRT *p*
Model 1—Reference model	GA at ECV + BMI + EFW + fetal position	0.645 (0.594–0.696)	Reference			
Model 2—Final model	DVP	0.714 (0.665–0.763)	0.069	0.002	22.73 (1)	<0.001
Model 3—Sensitivity	Previous CS	0.717 (0.669–0.766)	0.003	0.482	0.68 (1)	0.408
Model 4—Sensitivity	Placental location	0.724 (0.674–0.773)	0.006	0.502	4.92 (3)	0.178

## Data Availability

The raw data supporting the conclusions of this article will be made available by the authors on request due to privacy and ethical restrictions related to the use of retrospective clinical patient data.

## Supplementary Materials

The following supporting information can be downloaded at https://www.mdpi.com/article/10.3390/jcm15166443/s1; Table S1: Complete coefficients of the final multivariable model for ECV-related complications; Table S2: Complete coefficients of the sensitivity models; Table S3: Internal validation of the final model for ECV-related complications.

## Abbreviations

The following abbreviations are used in this manuscript:

AUC	Area under the receiver operating characteristic curve
ECV	External cephalic version
