# Supplementary material for: Development and Internal Validation of a Multivariable Prediction Model for Complications After External Cephalic Version in Singleton Term Pregnancies
Source: J Clin Med. 2026 Aug 20;15(16):6443. doi: 10.3390/jcm15166443 (PMC13514325; doi:10.3390/jcm15166443)
Supplement: Supplementary file 1 [file jcm-15-06443-s001.zip › Table_S1.pdf]

Supplementary Table S1. Complete coefficients of the final multivariable model for ECV-related complications

Predictor	β coefficient	Standard error	Adjusted OR (95% CI)	*p*
Gestational age at ECV, per week	0.0747	0.1978	1.08 (0.72–1.56)	0.705
Maternal BMI, per kg/m²	-0.0869	0.0272	0.92 (0.87–0.97)	0.001
Estimated fetal weight, per 100 g	-0.0002	0.0311	1.00 (0.94–1.06)	0.994
Fetal presentation: Transverse lie vs breech	-1.4450	0.7338	0.24 (0.04–0.79)	0.049
Deepest vertical pocket, per 10 mm	-0.3697	0.0830	0.69 (0.58–0.81)	<0.001

The final model was fitted in 876 complete cases including 97 ECV-related complications. Breech presentation was the reference category. Estimated fetal weight was modelled per 100-g increase and deepest vertical pocket per 10-mm increase.
Prediction equation: $LP = -0.794056 + 0.074732 \times GA\_ECV - 0.086933 \times BMI - 0.000219 \times (EFW/100) - 1.445023 \times Transverse\ Lie - 0.369740 \times (DVP/10)$.Transverse lie was coded as 1 for transverse lie and 0 for breech presentation. Predicted probability was calculated as $exp(LP)/[1 + exp(LP)]$.
