# Supplementary material for: Development and Internal Validation of a Multivariable Prediction Model for Complications After External Cephalic Version in Singleton Term Pregnancies
Source: J Clin Med. 2026 Aug 20;15(16):6443. doi: 10.3390/jcm15166443 (PMC13514325; doi:10.3390/jcm15166443)
Supplement: Supplementary file 1 [file jcm-15-06443-s001.zip › Table_S2.pdf]

Supplementary Table S2. Complete coefficients of the sensitivity models

Model	Predictor	β coefficient	Standard error	Adjusted OR (95% CI)	*p*
Model 3	Intercept	-0.6854	7.2646	–	0.925
	Gestational age at ECV, per week	0.0729	0.1982	1.08 (0.72–1.56)	0.713
	Maternal BMI, per kg/m²	-0.0871	0.0272	0.92 (0.87–0.97)	0.001
	Estimated fetal weight, per 100 g	-0.0014	0.0313	1.00 (0.94–1.06)	0.963
	Fetal presentation: Transverse lie vs breech	-1.4621	0.7345	0.23 (0.04–0.77)	0.047
	Deepest vertical pocket, per 10 mm	-0.3746	0.0833	0.69 (0.58–0.81)	<0.001
	Previous cesarean delivery	0.4405	0.5102	1.55 (0.51–3.91)	0.388
Model 4	Intercept	-0.3284	7.2445	–	0.964
	Gestational age at ECV, per week	0.0727	0.1974	1.08 (0.72–1.56)	0.713
	Maternal BMI, per kg/m²	-0.0876	0.0272	0.92 (0.87–0.96)	0.001
	Estimated fetal weight, per 100 g	-0.0080	0.0314	0.99 (0.93–1.06)	0.800
	Fetal presentation: Transverse lie vs breech	-1.4793	0.7356	0.23 (0.04–0.76)	0.044
	Deepest vertical pocket, per 10 mm	-0.3780	0.0837	0.69 (0.58–0.80)	<0.001
	Previous cesarean delivery	0.4409	0.5135	1.55 (0.51–3.94)	0.391
	Placental location: Posterior vs anterior	-0.4572	0.2499	0.63 (0.38–1.02)	0.067
	Placental location: Uterine fundus vs anterior	0.5836	0.5935	1.79 (0.49–5.30)	0.325
	Placental location: Lateral wall vs anterior	-0.0839	0.4400	0.92 (0.36–2.06)	0.849

Model 3 consisted of the final Model 2 plus previous cesarean delivery. Model 4 additionally included placental location. Breech presentation, no previous cesarean delivery, and anterior placental location were the reference categories. Estimated fetal weight was modelled per 100-g increase and deepest vertical pocket per 10-mm increase.
