# Supplementary material for: Development and Internal Validation of a Multivariable Prediction Model for Complications After External Cephalic Version in Singleton Term Pregnancies
Source: J Clin Med. 2026 Aug 20;15(16):6443. doi: 10.3390/jcm15166443 (PMC13514325; doi:10.3390/jcm15166443)
Supplement: Supplementary file 1 [file jcm-15-06443-s001.zip › Table_S3.pdf]

Supplementary Table S3. Internal validation of the final model for ECV-related complications

Performance measure	Ideal value	Apparent performance	Estimated optimism	Optimism-corrected performance
AUC	–	0.714 (0.665–0.763)	0.015	0.699
Calibration intercept	0	0.000	0.308	-0.308
Calibration slope	1	1.000	0.164	0.836
Brier score	0	0.094	-0.001	0.095

Internal validation was performed using 1000 bootstrap resamples. The apparent AUC is presented with its 95% confidence interval. Optimism-corrected estimates were obtained from 1000 bootstrap resamples without variable selection. A calibration intercept of 0 and a calibration slope of 1 represent ideal calibration. Lower Brier scores indicate lower overall prediction error.
